# 1249. Metabolomic Profile of Heterogeneous Vancomycin-Intermediate *Staphylococcus aureus* (hVISA) in Latin-American MRSA Isolates

**DOI:** 10.1093/ofid/ofab466.1441

**Published:** 2021-12-04

**Authors:** Betsy E Castro-Cardozo, Monica Cala, Catalina Espitia-Acero, Rafael Rios, Lizeth Leon, Cesar A Arias, Sandra Rincon, Jinnethe Reyes, Lorena Diaz, Lorena Diaz

**Affiliations:** 1 Molecular Genetics and Antimicrobial Resistance Unit and International Center for Microbial Genomics, Universidad El Bosque, Bogota, Colombia, Bogota, Distrito Capital de Bogota, Colombia; 2 Universidad de los Andes, Bogota, Cundinamarca, Colombia; 3 Universidad el Bosque, Bogota, Distrito Capital de Bogota, Colombia; 4 Universidad El Bosque, Bogota, Distrito Capital de Bogota, Colombia; 5 CARMiG, UTHealth and Center for Infectious Diseases, UTHealth School of Public Health, HOU, TX ; Molecular Genetics and Antimicrobial Resistance Unit and International Center for Microbial Genomics, Universidad El Bosque, BOG, COL, Houston, Texas

## Abstract

**Background:**

Vancomycin (VAN) is a first-line therapeutic option in severe infections caused by MRSA in Latin-America (LA). Development of reduced susceptibility to VAN has been associated with multiple changes in genes encoding pathways for cell wall metabolism and envelope stress responses. Nevertheless, a detailed and coherent mechanistic model to explain the phenotype remains elusive. To gain further insights into the hVISA phenotype, we sought to explore the metabolomic profile of hVISA isolates from LA.

**Methods:**

The undirected profile of intracellular *S. aureus* metabolites was analysed in four clinical isolates (two hVISA and two VSSA [Vancomycin susceptible *S. aureus*]) belonging to the Chilean/Cordobes clone-ST5 (the predominant hVISA lineage in LA), and two reference strains Mu3 and N315. The metabolites were obtained in mid-exponential growth phase in trypticase soy broth in five independent replicates. The metabolites were determined by reverse phase liquid chromatography and hydrophilic interaction. The metabolic profile was determined by variable importance in the projection score (VIP > 1). The differences between hVISA and VSSA were maximized by orthogonal partial least squares discriminant analysis (OPLS-DA) and the affected metabolic pathways were identified with MetaboAnalyst.

**Results:**

Among the differences identified in the metabolic profiles of hVISA respect to VSSA, 69 metabolites were relevant. Of these, 47 were fatty acids (including glycerol), 7 amino acids and 6 nucleosides (Table 1). These changes mainly impact the biosynthesis of amino acids derived from pyruvate since tyrosine, valine and leucine, had a reduction of 34%, 57% and 41% in hVISA compared to VSSA, respectively, which suggests alterations of the acid cycle tricarboxylic (TCA). Additionally, a reduction in purine and pyrimidine metabolism in hVISA was identified with reduction of nucleosides and dinucleotides derived from the pentose phosphate pathway.

Table 1. Metabolites with higher VIP scores in the comparison of the hVISA and VSSA profile

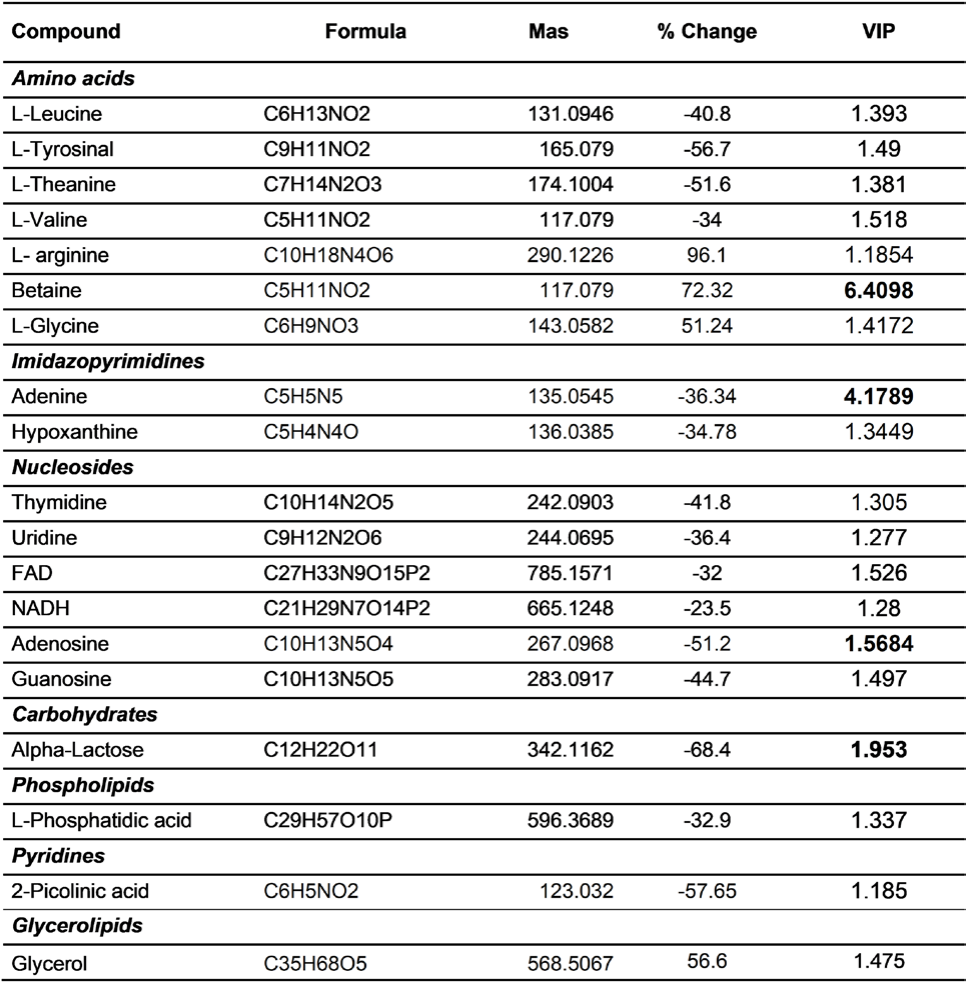

**Conclusion:**

We were able to observe metabolic alterations in TCA, pentose phosphate pathway and purine intermediates in hVISA-ST5 isolates. Our results support that gluconeogenesis and biosynthesis of carbohydrates and nucleic acids are the main pathways involved in the reduced susceptibility to VAN as reported in VISA isolates.

**Disclosures:**

**Cesar A. Arias, M.D., MSc, Ph.D., FIDSA**, **Entasis Therapeutics** (Grant/Research Support)**MeMed Diagnostics** (Grant/Research Support)**Merk** (Grant/Research Support) **Lorena Diaz, PhD** , Nothing to disclose

